# Surface and Interface Engineering in Integrated Photonic Sensors: Performance Trade-Offs, Stability, and Benchmarking

**DOI:** 10.3390/mi17050522

**Published:** 2026-04-25

**Authors:** Nikolay L. Kazanskiy, Dmitry V. Nesterenko, Svetlana N. Khonina

**Affiliations:** 1 Image Processing Systems Institute, NRC “Kurchatov Institute”, 151 Molodogvardeyskaya, Samara 443001, Russia; nesterenko@ssau.ru (D.V.N.); khonina@ipsiras.ru (S.N.K.); 2 Samara National Research University, 34 Moskovskoye Shosse, Samara 443086, Russia

**Keywords:** integrated photonic sensors, surface and interface engineering, optical sensing, surface functionalization, light–matter interaction

## Abstract

Surface and interface engineering has become a decisive factor in determining the performance and reliability of integrated photonic sensors. As photonic device architectures advance and geometric optimization strategies approach their fundamental performance limits, the nanoscale interface region where confined optical modes interact with the surrounding environment progressively becomes the dominant factor governing sensitivity, noise characteristics, and long-term operational stability. This review critically examines recent advances in these strategies applied to integrated photonic sensing platforms, including waveguide, interferometric, and resonant architectures. Emphasis is placed on how functional layers, nanomaterials, and hybrid interfaces modify light–matter interactions, while simultaneously introducing optical loss, spectral distortion, and stability constraints. Beyond summarizing reported sensitivity enhancements, this review analyzes performance benchmarking methodologies and highlights the limitations of conventional metrics such as bulk sensitivity and nominal limit of detection. Normalized figures of merit are discussed as essential tools for isolating genuine interface contributions across diverse platforms. Experimentally documented trade-offs between enhanced surface interaction, optical degradation, and temporal drift are examined in detail, alongside challenges related to reproducibility, wafer-scale variability, and long-term interface stability. By synthesizing insights from photonics, surface chemistry, and materials science, this review outlines key open questions and identifies design principles necessary for translating surface-engineered photonic sensors from laboratory demonstrations to robust and scalable sensing technologies.

## 1. Introduction

Integrated photonic sensors have become a cornerstone of modern optical sensing technologies, driven by the demand for compact, highly sensitive, and scalable platforms capable of addressing challenges in chemical analysis, biosensing, environmental monitoring, and healthcare diagnostics [1]. By confining and guiding light-on-chip-scale waveguides, these systems enable strong light–matter interactions within a small footprint while maintaining compatibility with complementary metal-oxide-semiconductor (CMOS) fabrication processes [2,3,4]. The sensing mechanism typically relies on monitoring changes in optical properties such as phase, resonance wavelength, or intensity that arise from perturbations in the surrounding environment [5,6,7,8,9]. As a result, integrated photonic sensors offer inherent advantages over bulk optical systems, including reduced sample volumes, high integration density, and the potential for large-scale, low-cost manufacturing [10].

A defining feature of integrated photonic sensors is the role of evanescent optical fields that extend beyond the waveguide core into the surrounding medium [11,12,13]. The resonance properties of optical modes are directly affected by analytes located at or near the sensor surface that provide the basis for label-free detection [14]. Variations in refractive index, molecular binding events, or changes in absorption at the interface translate into measurable optical signals. Over the past decade, substantial progress has been achieved through innovations in photonic architectures such as ring resonators [15], interferometers [16], photonic crystal cavities [17,18], and slot waveguides [19], each offering distinct trade-offs between sensitivity, noise influence, and long-term robustness. However, as these architectures have approached fabrication performance limits, further gains up to theoretical performance have become increasingly dependent on factors beyond purely optical design.

In this context, surface and interface engineering has emerged as a critical determinant of sensor performance. The interface between a resonance structure and the external environment governs not only the strength of light–matter interactions but also the selectivity, stability, and temporal response of the sensor [20,21]. Properties such as surface roughness, chemical composition, functional layer thickness, and interfacial uniformity directly influence optical loss, noise, and reproducibility [22]. While photonic design defines how light is confined and propagated, it is the engineered surface that dictates the selectivity efficiency of probing chemical and biological processes [23,24]. Consequently, advances in sensing performance are increasingly driven by technologies that tailor the interface at the molecular and nanoscale levels [25].

Surface engineering enables functionalities that are otherwise inaccessible to bare photonic platforms [26]. Through chemical functionalization, specific binding interactions can be introduced, transforming inherently non-selective refractive index sensors into highly selective detection systems [27]. Functional coatings, self-assembled monolayers, polymers, and biomolecular receptors allow molecular recognition events to be transduced into measurable optical characteristics with high specificity [28]. At the same time, emerging material systems such as two-dimensional materials, nanostructured oxides, and hybrid organic–inorganic interfaces offer new mechanisms for enhancing sensitivity through charge transfer, enhanced adsorption, and modified optical absorption [29]. These developments allow sensing interfaces to evolve from a passive boundary to an active component of the photonic device.

Along with these advantages, surface and interface engineering introduce new challenges that must be addressed for practical deployment [30]. Functional layers and advanced materials can increase optical loss, reduce quality factors in resonant devices, and introduce variability across fabrication batches [31]. Long-term stability under environmental exposure, resistance to fouling, and compatibility with standard microfabrication processes remain significant concerns [32]. Achieving enhanced surface interaction while maintaining acceptable optical performance requires a comprehensive understanding of both photonic and interfacial phenomena. This requirement highlights the importance of an integrated design framework in which surface chemistry, materials science, and photonic engineering are co-optimized rather than addressed independently [33].

Despite the extensive literature on integrated photonic sensors and surface functionalization, existing reviews typically treat photonic architectures and interface engineering separately. This review provides a unified, interface-centric perspective that explicitly links nanoscale interfacial properties with optical performance trade-offs, including optical loss, noise, and long-term stability. Particular emphasis is placed on benchmarking methodologies, highlighting the limitations of conventional sensitivity metrics and the need for normalized, noise-aware figures of merit for meaningful cross-platform comparison. By integrating insights from photonics, materials science, and surface chemistry, this work establishes design principles for scalable, reproducible, and reliable photonic sensing platforms.

**Figure 1:**
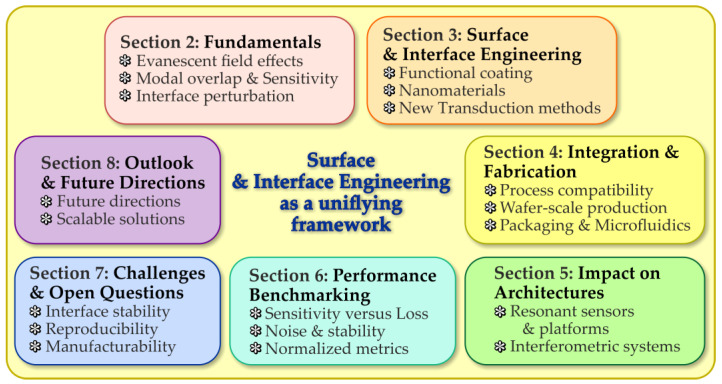
Schematic overview of the structure of this review, highlighting surface and interface engineering as a unifying framework for integrated photonic sensors. The two-column layout summarizes the progression from fundamental principles and interface engineering strategies to architectural impact, performance benchmarking, challenges, and outlook, emphasizing key trade-offs among sensitivity, loss, stability, and manufacturability.

The remainder of this review is organized as follows. Section 2 introduces the fundamental physical principles underlying surface-sensitive integrated photonic sensing, with emphasis on evanescent-field interactions, modal overlap, and interface-induced perturbations. Section 3 reviews the main surface and interface engineering strategies employed to enhance sensitivity and selectivity, including chemical functionalization, nanomaterial integration, and hybrid interface designs, and discusses their associated benefits and limitations. Section 4 addresses practical integration and fabrication considerations, highlighting process compatibility, wafer-scale reproducibility, and packaging constraints that critically influence sensor performance. Section 5 examines how these engineered interfaces impact different photonic sensor architectures, including waveguide-based, resonant, and interferometric platforms. Section 6 discusses performance benchmarking methodologies and trade-offs, emphasizing normalized and noise-aware figures of merit. Section 7 identifies key challenges and open questions related to stability, reproducibility, and manufacturability, followed by an outlook in Section 8 that outlines future research directions. Finally, Section 9 summarizes the main conclusions of the review. To guide the reader, Figure 1 provides a schematic overview of the structure of this review and the logical progression of the topics discussed.

## 2. Fundamentals of Surface-Sensitive Integrated Photonic Sensing

**Figure 2:**
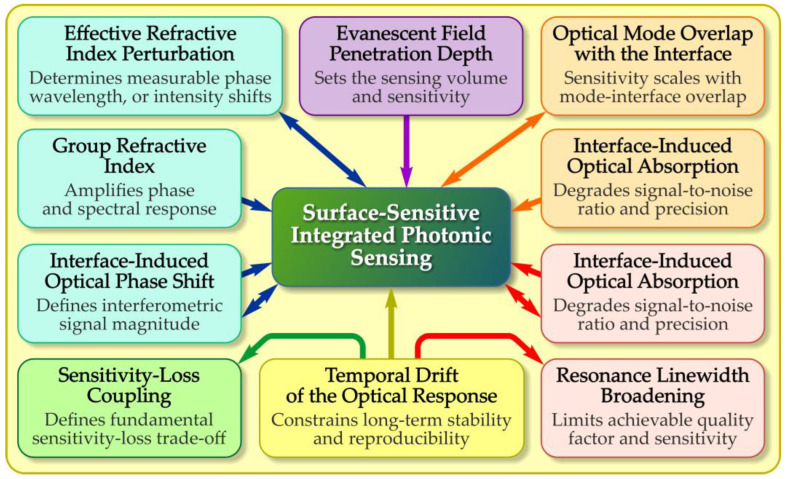
Interface-controlled parameters governing surface-sensitive integrated photonic sensing. Surface and interfacial perturbations modify the guided optical mode, enabling transduction of molecular interactions into optical signals, while optical losses and temporal drift limit sensitivity, stability, and reproducibility.

The fundamental operation of surface-sensitive integrated photonic sensors is based on the behavior of guided optical modes confined within high refractive index contrast waveguides and their interaction with the surrounding medium through evanescent fields [34,35]. When light propagates along an integrated waveguide, a fraction of the optical mode extends beyond the physical boundary of the core into the cladding or external environment. This evanescent field decays exponentially with distance from the surface, and its penetration depth is governed by the refractive index contrast between the waveguide and its surroundings, the modal polarization, and the operating wavelength. Any perturbation within this near-field region can modify the effective refractive index of the guided mode, forming the physical basis for surface-sensitive detection [36]. The performance of surface-sensitive integrated photonic sensors is fundamentally governed by interface-induced changes in optical characteristics, as summarized in Figure 2.

Surface-induced perturbations arise from a variety of mechanisms, including molecular adsorption, changes in local refractive index, surface mass loading, and absorption introduced by bound species or functional layers [37,38]. These perturbations translate into measurable optical characteristics such as resonance wavelength shifts in resonant structures, phase changes in interferometric configurations, or intensity variations in waveguide transmission. The magnitude of the optical response depends on the mode reshaping and replacement caused by the changes in the perturbation region, making surface functionalization and interface quality critical parameters [39]. Consequently, surface sensitivity cannot be regarded as an intrinsic material constant but rather as an emergent characteristic determined by the interplay between the optical mode distribution and the properties of the interface [40]. In this review, surface sensitivity is defined as the resonance shift (or phase shift) per unit refractive index change occurring within a surface-bound perturbation layer of finite thickness in the evanescent field region.

Waveguide geometry plays a central role in determining the local field enhancement. Shallow etched rib waveguides typically offer lower surface sensitivity but improved robustness and reduced scattering loss [41], whereas strip waveguides [42], slot waveguides [43], and suspended or exposed core designs [44] provide enhanced field penetration at the expense of increased sensitivity to surface imperfections. Similarly, higher index contrast platforms such as silicon on insulator enable tighter confinement but require deliberate geometrical or material solutions to extend sufficient optical energy into the sensing region [45,46]. These trade-offs highlight the inherent relationship between photonic design and surface sensitivity that underpins all integrated photonic sensing platforms.

The existence of a functionalized layer at the surface introduces additional complexity beyond detection of refractive index changes. Surface roughness at the nanometer scale can induce scattering losses that degrade the signal-to-noise ratio, particularly in resonant devices with high quality factors [47]. Chemical heterogeneity and non-uniform functional layer coverage lead to spatially varying perturbations, broadening resonances, and reducing measurement precision [48,49]. The thickness and optical properties of surface coatings further modify the modal distribution, potentially shifting the effective sensing region away from the target analytes if not carefully engineered [50,51]. Consequently, the sensing interface acts as both a transduction medium and a potential source of optical degradation [52].

Temporal response in surface-sensitive photonic sensors is also governed by interfacial phenomena [53]. The kinetics of analyte transport to the surface, adsorption and desorption rates, and diffusion through functional layers all influence response time and recovery behaviour [54]. While photonic structures can respond on ultrafast timescales, the overall sensor dynamics are often limited by surface processes rather than optical readout. This distinction underscores the importance of interface design not only for sensitivity and selectivity but also for achieving application-relevant response speeds.

Noise and stability considerations further emphasize the role of the interface [55]. Fluctuations in temperature, humidity, and nonspecific adsorption events can introduce spurious refractive index changes within the evanescent field region [56]. These effects are amplified in highly surface-sensitive configurations, leading to baseline drift and reduced long-term reliability [57,58]. Understanding the interplay between optical confinement, surface interaction strength, and environmental susceptibility is therefore essential for interpreting sensor signals and designing mitigation strategies [59]. Collectively, the fundamental principles of surface-sensitive integrated photonic sensing demonstrate that device performance arises from a strongly coupled system of factors in which optics of guided optical modes and processes at engineered interfaces must be considered simultaneously. Optical sensitivity originates from the dependence of the field propagation characteristics on the matter properties, whereas practical sensing performance is limited by optical loss, noise sources, and interfacial stability [60,61,62]. This conceptual framework establishes the basis for evaluating surface and interface engineering strategies designed to enhance light–matter interaction while maintaining optical integrity, forming the foundation for the subsequent sections of this review.

## 3. Surface and Interface Engineering Strategies

Surface functionalization is a central strategy for imparting chemical selectivity and enhancing sensitivity in integrated photonic sensors [63]. This is commonly achieved through engineered interfacial layers such as self-assembled monolayers, polymer brushes, hydrogels, and immobilized biomolecular receptors that introduce specific binding sites for target analytes. Molecular recognition events occurring at these interfaces are transduced into optical signals via perturbations of the local refractive index, optical absorption, or scattering within the evanescent field of the guided mode [64]. As a result, thickness control, chemical robustness, and lateral uniformity of functional layers directly influence sensor reproducibility, baseline stability, and device-to-device variability. Reviews of silicon photonic biosensors emphasize that degradation, swelling, and incomplete surface coverage can produce drift and hysteresis, which motivates the use of surface chemistries that remain stable under operating, regeneration, and storage conditions [65,66].

**Figure 3:**
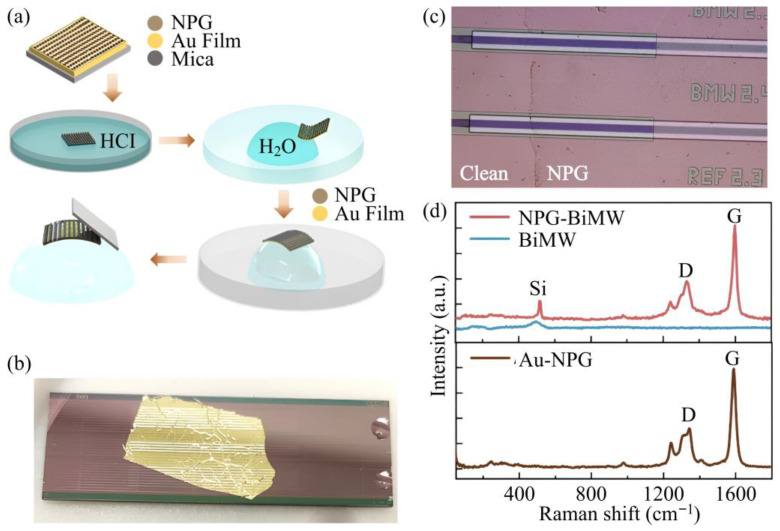
(**a**) Schematic of the polymer-free wet transfer used to integrate nanoporous graphene onto bimodal waveguide sensors. (**b**) Photograph of the device after transfer showing graphene coverage supported by a thin gold layer over the sensing region. (**c**) Optical micrograph after gold removal, where graphene coverage is identified by contrast differences. (**d**) Raman spectra of the bare waveguide, graphene integrated device, and growth substrate confirming the presence of nanoporous graphene [70].

Beyond conventional organic and biomolecular coatings, two-dimensional materials have emerged as a versatile class of engineered interfaces for photonic sensing platforms [67,68,69]. Their atomic-scale thickness, high surface-to-volume ratio, and strong light–matter interaction enable sensing mechanisms that extend beyond characterization of refractive index changes, including charge transfer, modulation of optical absorption, and enhanced surface adsorption. The integration of bottom-up synthesized nanoporous graphene on silicon nitride bimodal waveguide interferometric biosensors for C-reactive protein detection is presented [70]. In this study, Figure 3a–d are particularly instructive because they connect interface engineering to practical implementation and quality control. Figure 3a schematically outlines the transfer sequence used to place nanoporous graphene onto the sensing region, clarifying how the two-dimensional material is incorporated as a controlled process step rather than a post hoc coating. Figure 3b shows the resulting uniform film placement across the waveguide devices immediately after integration, supporting the central requirement that sensing interfaces must be laterally continuous over the optical interaction region. After removal of the auxiliary gold layer, Figure 3c uses optical microscopy to verify coverage and to identify any uncoated regions on the photonic surface, directly linking interface completeness to expected device-to-device repeatability. Finally, Figure 3d provides Raman spectra acquired at different stages, confirming that the nanoporous graphene signature is preserved through processing and remains present on the photonic chip after transfer. Together, these data define essential requirements for reliable integrated photonic interface engineering [70].

Moreover, hybrid interfaces that combine two-dimensional materials with polymers, oxides, or self-assembled monolayers provide additional flexibility in tailoring surface chemistry, mechanical integrity, and environmental stability, thereby expanding the accessible design space for photonic sensors [71,72]. More broadly, surface and interface engineering influences integrated photonic sensor performance through multiple, often competing physical mechanisms. In addition to enhancing the sensing selectivity, functional layers and advanced interfaces can modify optical loss, noise characteristics, long-term stability, and fabrication-induced variability [73]. Because these effects depend strongly on both the chosen interface strategy and the underlying photonic architecture, performance improvements achieved through surface modification may introduce trade-offs in other metrics. Table 1 summarizes the dominant sensing mechanisms, performance advantages, and associated limitations of commonly employed surface and interface engineering approaches, illustrating the need to co-optimize interface design and photonic architecture to achieve robust and scalable sensing performance.

While Table 1 summarizes the dominant interface engineering strategies and their qualitative trade-offs, a more rigorous comparison requires consideration of quantitative performance impacts reported across different studies. In particular, the effect of interface engineering on sensitivity must be evaluated together with the corresponding penalty in optical loss and noise. For example, SAMs, with thicknesses typically below 2–3 nm, introduce negligible additional propagation loss (<0.1–0.5 dB/cm) while enabling selective binding, but their contribution to sensitivity enhancement is inherently limited by the small interaction volume. In contrast, polymer and hydrogel coatings can increase effective analyte interaction thickness by one to two orders of magnitude, often resulting in sensitivity improvements of 2–10×, but at the cost of increased absorption and scattering losses that can exceed several dB/cm depending on thickness and material composition.

Similarly, ALD oxide overlayers primarily act to reduce surface roughness and scattering loss, with reported reductions in propagation loss exceeding an order of magnitude in optimized systems, although this is often accompanied by a measurable decrease in surface sensitivity due to reduced evanescent-field overlap. Two-dimensional materials and hybrid interfaces introduce additional transduction mechanisms, such as charge transfer and enhanced adsorption, which can lead to sensitivity enhancements beyond purely refractive-index-based detection; however, these gains are frequently offset by increased optical absorption and variability associated with material transfer and defect density.

Beyond individual performance metrics, it is also important to distinguish the technological maturity and scalability of these approaches. SAMs and ALD-based interfaces are generally compatible with CMOS fabrication and wafer-scale processing, making them suitable for scalable sensor platforms. Polymer coatings and hydrogel layers are widely used and offer high sensitivity, but their long-term stability and reproducibility remain application-dependent. In contrast, interfaces based on two-dimensional materials and complex hybrid structures are still largely at the proof-of-concept stage, where challenges related to uniformity, transfer-induced defects, and process integration limit their scalability.

This comparison highlights that the effectiveness of a given interface strategy cannot be assessed solely in terms of sensitivity enhancement but must be evaluated through a multi-parameter framework that includes optical loss, noise, stability, and manufacturability. Such a perspective enables a more realistic assessment of which approaches are suitable for practical deployment versus those that remain primarily exploratory.

Antibody-based surface functionalization is one of the most established routes for translating integrated photonic sensors into practical biosensing platforms, particularly for clinically relevant protein biomarkers and liquid-biopsy applications [85]. In these systems, antibodies immobilized at the sensing surface provide selective recognition, while the optical signal is generated through perturbation of the evanescent field in waveguide, resonant, plasmonic, or metasurface architectures. Representative implementations include microring-resonator immunosensors for human serum albumin detection [86], multiplexed ring-resonator platforms using antibodies immobilized on a copolymer layer for simultaneous biomarker detection [87], fiber-optic localized surface plasmon resonance immunoassays for thyroglobulin detection [88], slot-assisted metasurfaces for IgG sensing [89], and dielectric grating platforms for high-resolved near-field biosensing [90].

From an interface-engineering perspective, antibody functionalization introduces a distinct set of design constraints beyond those associated with generic organic or polymer coatings. Antibody layers are substantially thicker than self-assembled monolayers and therefore occupy a non-negligible fraction of the evanescent-field decay length, so their thickness, packing density, and orientation directly affect modal overlap with the target binding region and can partially dilute surface sensitivity even while improving capture specificity [86]. This trade-off is especially relevant in resonant and near-field-enhanced devices, where added biolayer thickness and interfacial nonuniformity can also increase optical loss, broaden spectral features, or reduce readout contrast [86,90].

The practical value of antibody functionalization, therefore, depends not only on recognition specificity but also on how immobilization chemistry preserves receptor activity and controls interfacial uniformity. In photonic biosensors, commonly used strategies include oxide-surface activation and silanization followed by covalent attachment, as well as polymer-assisted antibody immobilization and other schemes intended to improve receptor density and assay robustness [87,91]. These approaches can improve analytical performance, but they also raise familiar interface-related challenges, including denaturation during immobilization, degradation under regeneration conditions, nonspecific adsorption, and reduced long-term stability in clinically relevant media [85].

Taken together, these studies show that antibody-based biofunctionalization should be treated as a distinct interface-engineering regime rather than as a generic subclass of “biomolecular receptors”. For integrated photonic biosensors, performance is determined by the combined optimization of immobilization chemistry, biolayer thickness, receptor accessibility, and optical-mode confinement, and this is precisely where trade-offs between specificity, sensitivity, optical loss, and temporal stability become most application-critical [85,86,87].

## 4. Integration and Fabrication Considerations

Interface integration in photonic sensors is ultimately constrained by process compatibility, because the same surface that must support selective chemistry also has to survive wafer-scale microfabrication, packaging, and fluidic handling [92,93]. Table 2 highlights the interface integration processes that most strongly couple surface engineering to optical performance, yield, and scalability in integrated photonic sensors, emphasizing fabrication-relevant constraints rather than application-specific chemistries.

In practice, engineered interfaces are introduced by a variety of techniques such as oxygen plasma or UV ozone activation, silanization on SiO_2_ or Si_3_N_4_, polymer deposition by spin coating, and thin film growth by physical vapor deposition, chemical vapor deposition, or atomic layer deposition [94,95]. Each step changes not only surface chemistry but also optical performance through added absorption, stress-induced cracking, and thickness nonuniformity that shifts modal confinement and increases scattering loss. In addition to thickness and roughness effects, fabrication-induced interface properties such as surface damage, contamination, and process variability can significantly influence photonic device performance [96]. Plasma exposure, etching processes, and lithographic patterning can introduce defects, dangling bonds, and surface states that increase optical absorption and scattering, while also modifying adsorption behavior and long-term stability [97]. Residual contaminants from photoresists, transfer processes, or chemical treatments can further alter the local refractive index and introduce uncontrolled optical loss, particularly in surface-sensitive configurations [98]. Moreover, process-induced variability at the wafer scale, including nonuniform surface activation, incomplete functional layer coverage, and spatial fluctuations in interface quality, can lead to significant device-to-device variation in sensitivity, noise characteristics, and baseline drift. These effects highlight that interface properties are not solely defined by intentional surface engineering, but are also strongly shaped by fabrication history, necessitating process control and interface-aware metrology to ensure reproducible photonic sensor performance [99,100].

For ultra-surface-sensitive devices, nm scale variability in functional layer thickness or surface roughness can measurably broaden resonances and reduce quality factor, creating a tight coupling between chemical processing windows and photonic figures of merit [101]. This coupling can be further understood by considering how nanometer-scale variations in interface properties propagate into sensing observables. For instance, a ±1 nm variation in functional layer thickness modifies the effective refractive index of the guided mode, leading to measurable uncertainty in resonance wavelength shifts in resonant sensors and phase fluctuations in interferometric configurations. In high-Q resonators, such variations can produce resonance shifts comparable to or exceeding the intrinsic linewidth, thereby degrading the achievable LOD due to the coupling between linewidth and noise [102]. Similarly, in interferometric systems, small variations in effective index accumulate over the interaction length and manifest as phase noise, contributing to baseline fluctuations and long-term drift. These effects illustrate that fabrication-induced variability does not merely affect optical loss but directly sets the uncertainty floor of sensing metrics such as LOD, signal-to-noise ratio, and temporal stability.

It is also important to distinguish between laboratory-scale reproducibility and foundry-level reproducibility. Laboratory demonstrations typically rely on controlled, small-batch processing conditions, where surface functionalization and interface quality can be optimized for individual devices. In contrast, wafer-scale fabrication introduces additional variability sources, including spatial nonuniformity across the wafer, process drift between runs, and statistical fluctuations in surface activation and layer deposition. As a result, variability in sensing performance at the foundry level is often dominated by process-induced dispersion in interface properties rather than by intrinsic device design, highlighting the need for process-tolerant interface strategies and standardized metrology for large-scale deployment.

This sensitivity to fabrication-induced surface quality is further highlighted by emerging additive fabrication approaches such as two-photon polymerization (TPP), which enable three-dimensional fabrication of complex micro- and nanophotonic structures with submicrometer resolution, offering new opportunities for integrated optics, microresonators, and freeform waveguide geometries [103]. However, the layer-by-layer polymerization process and voxel-based exposure inherently introduce nanoscale surface roughness and structural inhomogeneity, which can significantly increase optical scattering losses and degrade quality factors in photonic devices [104,105]. In contrast to planar CMOS-compatible fabrication, where interface roughness can be minimized through optimized deposition and etching, TPP-fabricated structures often require additional post-processing steps such as thermal reflow, chemical smoothing, or conformal coating to improve optical performance. These challenges highlight that, despite its geometric flexibility, TPP remains strongly constrained by interface-induced optical loss, reinforcing the broader principle that fabrication-induced surface quality is a dominant factor in determining photonic device performance.

**Figure 4:**
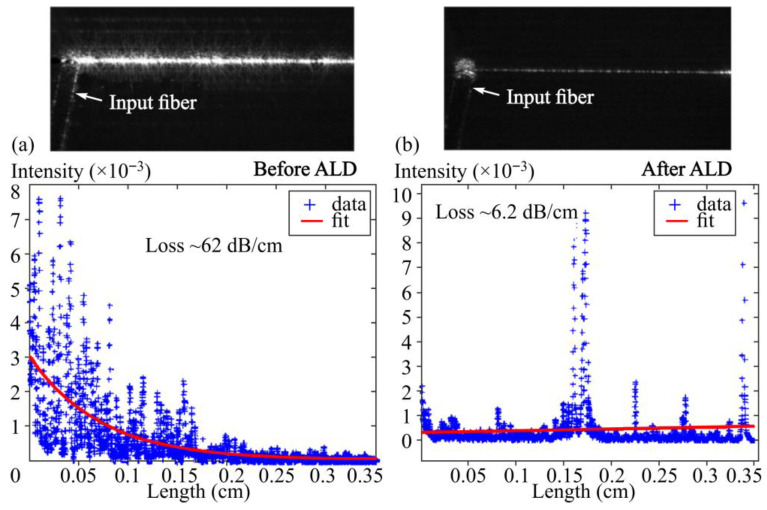
(**a**,**b**) Optical microscopy images of silicon nitride strip waveguides excited via grating couplers, together with extracted optical intensity profiles used to quantify propagation loss. The uncoated, air-clad waveguide exhibits extremely high attenuation of about 60 dB/cm, whereas the same structure after conformal deposition of a 40 nm Al_2_O_3_ layer by atomic layer deposition shows a pronounced reduction in loss to approximately 6 dB/cm. The comparison highlights the strong influence of nanometer-scale interface engineering on optical performance [106].

In planar CMOS-compatible platforms, this coupling is experimentally illustrated by Khanna et al., where atomic layer deposition of an Al_2_O_3_ overlayer was used to passivate Si_3_N_4_ waveguides fabricated in a CMOS pilot line [106]. As shown in Figure 4a,b, optical intensity decay measurements before and after ALD coating reveal a dramatic reduction in propagation loss, directly linking nanometer-scale interface smoothing to improved optical performance. The results demonstrate that interface engineering steps intended for surface stabilization or functionalization can dominate device loss and reproducibility, underscoring the need for metrology-driven control of film thickness, refractive index, roughness, and contamination, as well as fabrication flows that minimize residues from photoresists and transfer media [106].

Manufacturability also depends on how post-fabrication steps interact with lithography and packaging [107]. Solvents, developers, and lift-off chemicals can disrupt biofunctional layers, while plasma cleaning that improves wettability can also alter surface termination and introduce charge traps that affect adsorption behavior and drift. Likewise, integrating microfluidics introduces additional constraints, including bonding strength, dead volume, and alignment tolerance, while maintaining optical access and protecting functionalized areas from thermal and mechanical damage [108]. Wafer-level integration approaches are increasingly favored because they reduce device-to-device variability and enable parallelization, but they demand chemical treatment and deposition methods that are compatible with standard pilot-line processes [109]. Collectively, these studies indicate that surface interfaces are strongly coupled to fabrication and packaging processes, with constraints imposed by thermal impacts, solvent compatibility, and plasma exposure [79,93,110,111]. As a result, long-term stability, yield, and calibration behavior are often governed by limitations introduced during surface preparation and integration rather than by the sensing mechanism alone.

**Figure 5:**
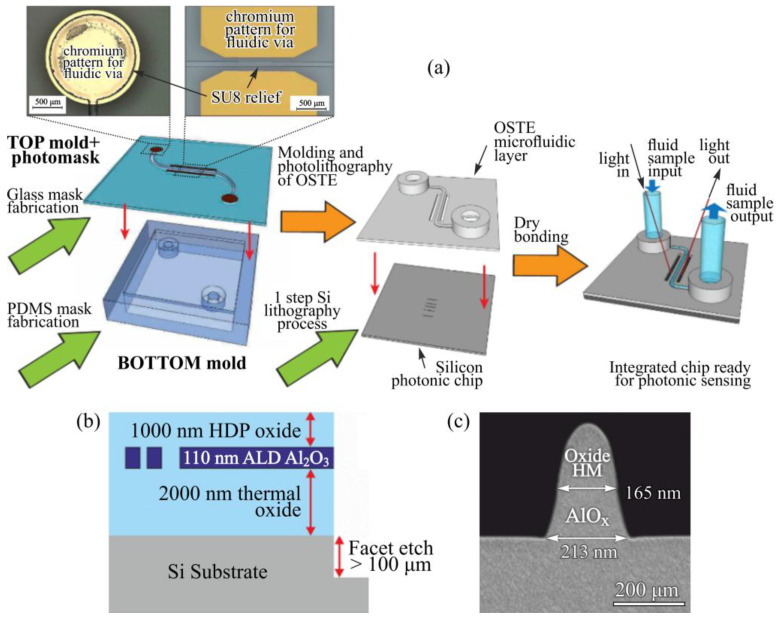
(**a**) Schematic overview of wafer-level microfluidic integration with photonic sensor chips. A patterned glass mask incorporating metal features via definition and photoresist relief structures for channel formation is used as a combined lithography and molding template. A secondary elastomer mold defines the chip perimeter and fluidic interconnects. Following ultraviolet polymerization, development, and dry bonding to the photonic substrate, the assembled device forms a compact optofluidic sensing platform [112]. (**b**) Cross-sectional illustration of integrated Al_2_O_3_ photonic waveguides fabricated using CMOS-compatible thin-film deposition, showing the layered waveguide stack and cladding structure [115]. (**c**) SEM image of an Al_2_O_3_ waveguide cross section with a nominal thickness of 110 nm and a critical dimension of 150 nm, highlighting sidewall definition and interface quality [115].

Concrete examples illustrate these constraints and solutions. Errando-Herranz et al. demonstrated a wafer-level method for integrating polymer microfluidics with grating-coupled silicon photonic sensors while addressing robust bonding to biofunctionalized surfaces [112]. As illustrated in Figure 5a, their approach combines photopatterned optical and fluidic vias with molded microchannels in an off-stoichiometry thiol-ene polymer, enabling precise alignment between photonic elements and microfluidic interfaces while preserving surface chemistry. This figure highlights packaging and fluid handling as dominant integration bottlenecks for scalable biosensing, emphasizing that optical performance, interface functionalization, and mechanical assembly must be co-designed rather than treated as independent steps [112]. Henriksson et al. reviewed strategies for functionalizing oxide-free silicon using robust Si–C-bonded interlayers, which are directly relevant when silicon surfaces are used as the chemical interface and long-term stability is needed beyond conventional silane layers [113]. Steglich et al. reviewed silicon-on-insulator ring resonator biosensors and discussed how practical device performance is shaped by fabrication tolerances, loss mechanisms, and surface preparation, reinforcing the idea that interface processes must be co-designed with resonator performance targets [114].

At the foundry scale, Neutens et al. reported low-loss Al_2_O_3_ waveguides fabricated in a 200 mm CMOS pilot line [115]. Figure 5b illustrates the wafer-scale layer stack and waveguide cross-section, highlighting the importance of conformal thin-film deposition, precise thickness control, and smooth interfaces for achieving low optical loss and reproducible modal confinement. Figure 5c further demonstrates uniformity and yield across the full wafer, underscoring that interface roughness, film stress, and process variability are critical considerations for manufacturing-relevant sensing platforms rather than purely laboratory-scale concerns. Finally, work integrating CVD graphene onto silicon nitride waveguides illustrates practical issues such as transfer residues, wrinkles, and added optical absorption, which are analogous to the challenges encountered when adding functional two-dimensional layers to sensing waveguides [80].

**Table 2:** Critical interface integration and fabrication considerations for integrated photonic sensors.

Integration Category	Representative Processes	Key Interface Parameters	Dominant Optical Consequences	Manufacturing Constraints	Representative Examples
Conformal dielectric interface engineering [116]	Atomic layer deposition (Al_2_O_3_, HfO_2_), PECVD oxides [117,118]	Roughness (<1 nm RMS), thickness (±1 nm), refractive index, film stress	Strong reduction in propagation loss; improved Q-factor; stabilized modal confinement	Thermal budget limits; precursor purity; stress-induced cracking	ALD passivation of Si_3_N_4_ waveguides showing >10× loss reduction; CMOS-scale Al_2_O_3_ waveguides [117]
Functional layer deposition & thickness control [119]	Silanization, polymer spin coating, hydrogel layers [120]	Layer thickness, swelling behavior, lateral uniformity	Resonance broadening; mode displacement; baseline drift	Sensitivity to humidity and solvents; nm scale thickness variability	Silicon photonic biosensor surface functionalization studies [64]
Two-dimensional material integration [68,81]	CVD graphene transfer, nanoporous graphene integration	Coverage continuity, wrinkles, residues, optical absorption	Added absorption loss; enhanced surface interaction; scattering from defects	Transfer residues; alignment over waveguides; yield	Nanoporous graphene on bimodal waveguides; CVD graphene on Si_3_N_4_
Lithography and process compatibility [121,122]	Photoresist patterning, plasma exposure, lift-off	Chemical contamination, surface termination	Increased scattering loss; adsorption hysteresis; drift	Biofunctional layer degradation; solvent incompatibility	Sensor-last fabrication flows in integrated photonics
Microfluidic and packaging integration [93,112,123]	Wafer-level bonding, thiol-ene or polymer microfluidics	Bonding strength, dead volume, surface accessibility	Optical access loss; stress-induced degradation	Thermal/mechanical damage; alignment tolerance	Wafer-level optofluidic integration of photonic sensors [109]
Wafer-scale manufacturability [124]	CMOS pilot-line deposition and patterning	Thickness uniformity, interface roughness, yield	Reproducible low loss; consistent device performance	Strict contamination control; statistical process variation	200 mm CMOS fabrication of low-loss photonic waveguides [115]

## 5. Impact of Surface Engineering on Sensor Architectures

The impact of surface and interface engineering on integrated photonic sensors is inherently architecture-dependent, because different photonic configurations balance surface-induced perturbations against distinct mechanisms of optical confinement, propagation loss, and signal transduction [125]. Surface modification does not merely introduce chemical selectivity; it alters the electromagnetic boundary conditions at the waveguide–environment interface, thereby modifying the effective refractive index, modal overlap with the analyte, and optical loss [126]. Consequently, identical surface chemistries or functional layers can lead to markedly different sensing performance when implemented in waveguide-based, resonant, or interferometric architectures.

### 5.1. Waveguide-Based Sensors: Surface Overlap Versus Propagation Loss

**Figure 6:**
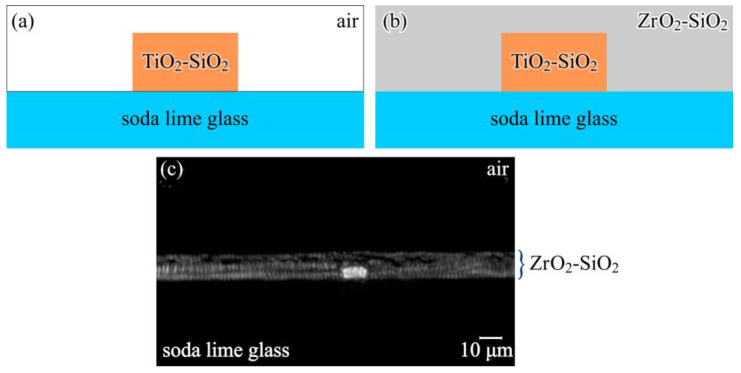
Schematic and experimental views of sol–gel waveguide configurations fabricated on a glass substrate. Panel (**a**) shows a TiO_2_–SiO_2_ waveguide core directly exposed to the surrounding medium, whereas panel (**b**) depicts the same core embedded within a ZrO_2_–SiO_2_ overlayer acting as a protective cladding. Panel (**c**) presents the experimentally observed guided optical mode at the output of the buried waveguide, confirming efficient light confinement in the encapsulated structure [129].

In straight waveguide sensors, the sensing signal originates from changes in the mode’s effective refractive index induced by perturbations within the evanescent field region, while the noise floor is largely governed by propagation loss and baseline stability [46,127]. Surface coatings, therefore, act simultaneously as functional recognition layers and optical boundary layers, with their thickness, refractive index, roughness, and absorption coefficient directly influencing modal confinement and attenuation [128]. Thin, low-index interfaces such as silane layers or self-assembled monolayers typically preserve low optical loss while enabling surface sensitivity. In contrast, thicker polymeric coatings or nanostructured layers can enhance analyte capture efficiency but often introduce additional absorption and scattering. Atomic-layer-deposited oxide interlayers are frequently employed to improve surface uniformity and chemical stability; however, they can also shift the modal maximum away from the sensing interface, leading to partial dilution of surface sensitivity [117]. These competing effects highlight the need for careful optimization of interface thickness and material composition in waveguide-based sensors. A representative example of this trade-off is provided by sol–gel waveguide sensors, where hybrid TiO_2_-SiO_2_ cores are fabricated using UV photolithography and direct laser writing to achieve low-loss light guidance over large areas [129]. As illustrated in Figure 6a, an exposed TiO_2_-SiO_2_ core surrounded by air offers increased interaction with the external environment but remains susceptible to surface-induced scattering and environmental perturbations. Encapsulating the core within a ZrO_2_-SiO_2_ cladding, as shown in Figure 6b, shifts the optical mode away from the interface, reducing evanescent-field overlap while significantly suppressing propagation loss and improving mechanical and thermal stability, consistent with the guided output observed in Figure 6c [129].

Slot waveguides and exposed-core geometries represent the opposite extreme of enhanced surface interaction [130]. By concentrating a large fraction of the optical energy in low-index regions, these architectures achieve strong evanescent-field overlap with the analyte but become highly sensitive to interface imperfections [131]. Nanometer-scale variations in coating thickness or roughness can induce significant excess loss and spectral noise, requiring tight coupling between surface engineering and fabrication control [132].

### 5.2. Resonant Architectures: Surface Sensitivity Constrained by Quality Factor

Resonant photonic sensors, including microring resonators and photonic crystal cavities [133], amplify surface-induced perturbations through high-quality factors and strong field localization, enabling detection of extremely small refractive-index changes or molecular binding events and making these architectures highly attractive for surface-engineered sensing [121,134]. At the same time, their performance is fundamentally constrained by surface and interface quality, as even minimal added loss can dominate the total cavity linewidth in high-Q devices [135]. This limitation is clearly demonstrated by experimental studies showing that nanoscale surface roughness and interface imperfections introduce excess scattering loss that broadens resonances and reduces the achievable quality factor [136].

**Figure 7:**
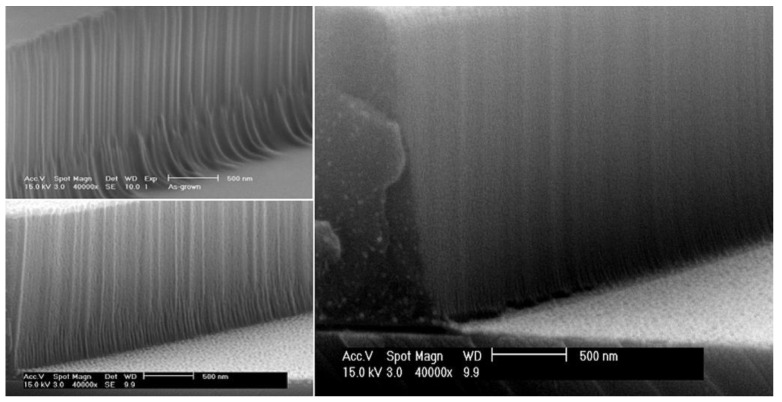
Scanning electron micrographs of polymer microring resonator sidewalls fabricated using a mold-based process, illustrating the effect of different post-fabrication treatments on interface smoothness. The image in the top left shows the as-fabricated sidewall without resist reflow, bottom left corresponds to a device after resist reflow, and on the right shows further surface smoothing achieved through the combination of resist reflow and subsequent thermal oxidation [136].

Systematic investigations of silicon microring resonators fabricated with different sidewall treatments reveal that untreated interfaces exhibit pronounced roughness, while resist reflow and subsequent thermal oxidation progressively smooth the sidewalls, as shown in Figure 7.

These structural improvements translate directly into reduced scattering loss and significantly narrower resonance linewidths, confirming that surface and interface quality, rather than resonator geometry alone, often sets the dominant limit on Q. Such findings underscore that functional layers, biomolecular films, or nanomaterial coatings can rapidly degrade resonant performance if they introduce absorption, refractive-index inhomogeneity, or spatial non-uniformity, potentially leading to resonance splitting and increased spectral noise [136]. Consequently, effective surface engineering for resonant photonic sensors requires ultrathin and highly uniform functional layers, controlled receptor densities, and selective modification confined to regions of high modal overlap while passivating non-sensing areas, so that enhanced surface sensitivity is achieved without compromising optical coherence and detection precision.

### 5.3. Interferometric Sensors: Phase-Based Robustness to Interface-Induced Loss

Interferometric architectures, such as Mach–Zehnder [137] and bimodal waveguide interferometers [70,138], transduce surface perturbations into optical phase shifts rather than relying on narrow resonance features. In these devices, refractive-index changes induced by surface modification or analyte binding in the sensing arm generate a differential phase delay relative to a reference arm, which is converted into a measurable intensity modulation at the output. This operating principle renders interferometric sensors more tolerant to moderate increases in propagation loss compared to high-Q resonant devices [83]. As a result, they offer greater flexibility in surface engineering, allowing the use of thicker functional layers, porous coatings, or hybrid interfaces without severe degradation of readout fidelity [139].

This behavior has been experimentally demonstrated in asymmetric Mach–Zehnder interferometric biosensors fabricated on silicon nitride platforms, where the sensing arm is locally exposed and chemically functionalized with antibody fragments for specific molecular recognition [140]. In these systems, surface modification is achieved by silanization of the exposed waveguide region followed by immobilization of Fab′ fragments, enabling selective analyte binding while preserving stable interferometric operation. The sensing signal arises from phase accumulation over a millimeter-scale interaction length rather than from strong optical confinement or narrow spectral features, allowing refractive-index changes induced by biomolecular binding to be detected with high precision even in the presence of additional surface layers. Real-time measurements performed under continuous liquid flow conditions demonstrate stable and repeatable phase responses, confirming that interferometric readout remains robust despite the introduction of functionalized interfaces and moderate optical attenuation. These characteristics highlight the suitability of interferometric architectures for complex surface chemistries and long-term biosensing applications where surface functionalization, regeneration, and environmental fluctuations are unavoidable.

**Figure 8:**
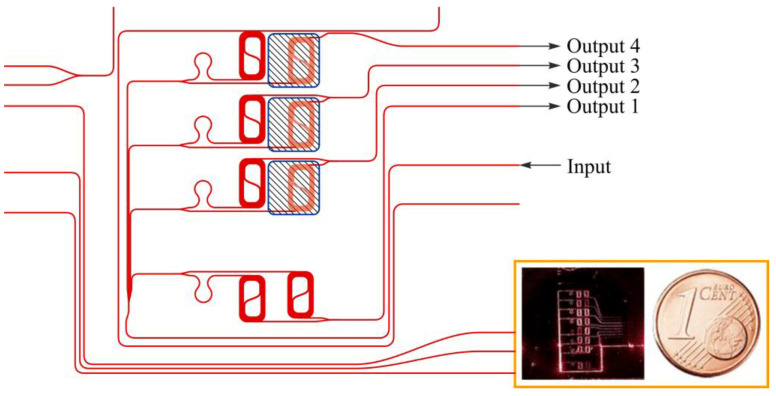
Schematic representation of the asymmetric Mach–Zehnder interferometric sensor layout used in this work. The inset shows an optical photograph of the fabricated chip, with a one-euro cent coin included to indicate the physical scale. The device integrates eight asymmetric Mach–Zehnder interferometers on a single chip [140].

Figure 8 illustrates a representative functionalized asymmetric Mach–Zehnder interferometric sensor architecture. The schematic shows multiple interferometers integrated on a single chip, with one arm of each interferometer locally exposed through a sensing window to enable evanescent-field interaction with the surrounding liquid environment, while the reference arm remains fully cladded to provide a stable optical baseline. The extended sensing arm length, implemented using a compact spiral geometry, enables cumulative phase accumulation and enhances sensitivity without relying on high-Q resonances. The inset photograph highlights the compact footprint and scalability of the interferometric platform, emphasizing its compatibility with integrated microfluidics and parallel sensing. Together, the architecture and experimental configuration illustrate how phase-based interferometric detection enables robust surface-sensitive sensing while maintaining tolerance to interface-induced optical loss in functionalized photonic biosensors [140].

### 5.4. Surface Engineering-Enabled Transition from Plasmonic to Dielectric Architectures

Ligand interactions with membrane proteins underpin a wide range of biological functions and therapeutic mechanisms, making their quantitative characterization essential for drug discovery and biomedical research [141,142]. Among label-free optical techniques, surface plasmon resonance has been widely adopted for probing molecular binding kinetics at interfaces due to its high surface sensitivity, real-time operation, and compatibility with complex biological environments [143,144].

Despite these advantages, conventional SPR relies on plasmon excitation in noble metal films, most commonly gold, which introduces intrinsic architectural limitations, including significant optical absorption loss, fluorescence quenching, and restricted compatibility with live-cell and multimodal optical measurements [36]. These material constraints can hinder applications involving membrane proteins and single-cell analysis.

To address these challenges, surface-sensitive dielectric waveguide-based approaches have been developed as alternatives or extensions to traditional SPR platforms [36]. By introducing additional dielectric layers onto standard SPR sensor substrates using scalable thin-film deposition methods, guided waveguide modes can be supported that maintain strong surface confinement while replacing the metal interface with biologically favorable materials such as silica. This modification preserves the essential benefits of SPR while enabling improved compatibility with cellular systems and reducing undesirable optical losses associated with metal absorption [36].

Dielectric waveguide-based sensing architectures typically exhibit narrower resonance features and enhanced surface electric fields compared to plasmonic counterparts [145], resulting in increased sensitivity and improved measurement precision [36]. When combined with advanced signal-processing schemes such as amplitude- or phase-modulated interrogation, these platforms can significantly outperform conventional SPR under identical optical interrogation conditions [146]. The resulting gains in precision enable measurements at the level of individual cells, facilitating investigations of cell-to-cell variability in ligand binding behavior, a phenomenon increasingly recognized as a contributor to therapeutic resistance [36]. From an architectural perspective, the integration of surface-sensitive dielectric waveguide modes into SPR-inspired platforms illustrates how surface and interface engineering can decouple surface sensitivity from the fundamental limitations imposed by metallic interfaces, thereby expanding the applicability of label-free biosensing technologies to biological and pharmaceutical studies that demand high precision, biocompatibility, and compatibility with live-cell analysis [36].

To provide a unified perspective, the architecture-dependent effects discussed above are here compared using a common set of performance metrics, including surface sensitivity, tolerance to optical loss, robustness to functional layers, and scalability. While waveguide-based sensors offer a balanced trade-off between sensitivity and loss, resonant architectures achieve the highest sensitivity due to strong field confinement but remain highly susceptible to interface-induced scattering and absorption. In contrast, interferometric sensors rely on phase accumulation over extended interaction lengths, resulting in greater tolerance to propagation loss and functional layers, albeit with increased footprint and sensitivity to environmental phase noise. This comparison clarifies that no single architecture is universally optimal, and that the effectiveness of surface and interface engineering must be evaluated within architecture-specific performance trade-offs.

## 6. Performance Benchmarking and Trade-Offs

Standardizing the evaluation of surface- and interface-engineered photonic sensors requires adopting performance metrics that reflect both intrinsic optical transduction and practical measurement conditions. Traditional reporting of bulk sensitivity and limit of detection (LOD) often conflates device scale, interrogation scheme, and readout noise, making cross-platform comparisons unreliable. Recent reviews of integrated photonic technologies emphasize the importance of metrics that normalize sensitivity by interaction length or optical loss to isolate the effect of interface design on device performance, particularly in waveguides, interferometers, and resonators, where functional layers alter propagation characteristics and modal confinement [147]. Moreover, analytical approaches that link LOD to quality factors and noise characteristics, such as defining LOD based on a signal change distinguishable from baseline fluctuations at a stated statistical confidence, provide a more robust basis for comparing devices under different operating conditions, independent of geometry or interrogation protocol. These normalized figures of merit help clarify when enhancements are due to true interface contributions rather than changes in device footprint or optical power.

A core challenge in benchmarking engineered photonic sensors is balancing enhanced surface interaction with the deleterious optical effects introduced by functional coatings and nanostructures. While increased evanescent-field overlap or high-index contrast layers can improve nominal sensitivity, they often introduce additional absorption and scattering that elevate background noise and broaden resonance linewidths, diminishing practical detectability and repeatability [64]. Detailed analyses of advanced sensor design underscore that these trade-offs are central to real-world performance, especially where stability and repeatability over time are critical for deployment outside controlled laboratory settings. For example, in photonic crystal and whispering-gallery-mode platforms, environmental drift and mechanical perturbations can dominate measurement noise even when intrinsic sensitivity is high, underscoring that high quality factors alone do not guarantee low LOD in practical use. Comprehensive benchmarking, therefore, incorporates temporal stability metrics, noise characterization over relevant timescales, and explicit reporting of operating conditions so that interface strategies delivering genuine performance improvements can be reliably identified.

To consolidate these considerations and provide a structured overview of current benchmarking practices, Table 3 summarizes commonly reported performance metrics alongside their key limitations and recommended normalized or noise-aware alternatives. The table highlights how different figures of merit capture complementary aspects of sensor performance, including sensitivity enhancement, optical degradation, noise behavior, and temporal stability. By explicitly linking each metric to representative open-access studies, the comparison clarifies how surface and interface engineering influences both transduction efficiency and practical detectability, and it provides a unified framework for evaluating trade-offs across diverse photonic sensing platforms.

While the metrics summarized above provide a conceptual framework for performance benchmarking, their practical implementation requires explicit experimental procedures and can significantly influence the interpretation of reported sensor performance. For example, two sensors exhibiting similar bulk sensitivity may demonstrate markedly different practical detectability when optical loss and noise are considered. A device with higher nominal sensitivity but increased propagation loss may ultimately yield a lower effective signal-to-noise ratio, resulting in a poorer noise-limited LOD compared to a lower-loss configuration. In this context, metrics such as sensitivity normalized by optical loss provide a more physically meaningful basis for cross-platform comparison.

From an experimental perspective, noise-limited LOD should be determined from measurable baseline fluctuations under realistic operating conditions rather than inferred solely from sensitivity. This requires time-series acquisition of the sensor output in the absence of an analyte to evaluate the standard deviation of the baseline signal. The choice of averaging time is critical, as short averaging intervals primarily capture high-frequency noise, whereas longer timescales reveal low-frequency drift and environmental fluctuations that directly impact practical detection limits. Temporal stability can be quantified using Allan deviation analysis, which evaluates signal variance as a function of averaging time and enables identification of dominant noise mechanisms, including white noise, flicker noise, and drift. This analysis directly defines the optimal integration time and provides a consistent basis for comparing stability across different sensor platforms.

## 7. Challenges and Open Questions

Despite substantial advances in surface and interface engineering, several experimentally documented challenges continue to limit the reliability and scalability of integrated photonic sensors. A dominant unresolved issue is the long-term physicochemical stability of functional interfaces under continuous optical interrogation and prolonged exposure to aqueous or chemically complex environments [165,166,167]. Studies have shown that commonly used silane-based monolayers and polymeric coatings undergo gradual hydrolysis, oxidation, and molecular rearrangement, in which alkoxysilane groups hydrolyze under trace ambient moisture and subsequently condense into evolving siloxane networks and partially interconnected inorganic clusters [168,169,170]. Such time-dependent interfacial evolution leads to effective refractive index drift and increased optical loss in surface-sensitive photonic sensors. In high surface-sensitivity configurations, even sub-nanometer variations in interface thickness or composition can produce measurable baseline drift and resonance broadening, raising fundamental questions about the achievable long-term precision of surface-engineered photonic sensors [101]. For emerging interfaces based on two-dimensional materials or hybrid organic–inorganic layers, additional concerns arise from defect-mediated adsorption, residual transfer contamination, and environmental doping effects, which can modify optical absorption and charge transfer pathways over time and remain insufficiently understood [72,171].

Reproducibility across devices and fabrication batches represents a second critical open challenge that directly impacts performance benchmarking and manufacturability. Experimental reports consistently demonstrate that variability in surface activation, functional layer coverage, and nanoscale roughness leads to significant spread in sensitivity, noise floor, and limit of detection, even for nominally identical photonic architectures fabricated on the same wafer [172]. This variability is amplified in resonant devices where spatial nonuniformity of surface functionalization can induce resonance splitting, excess scattering, and device-specific spectral features that complicate calibration and comparison [135]. While wafer-scale photonic fabrication has reached high maturity, equivalent standardization in surface functionalization and interfacial metrology remains lacking [173,174]. Open questions, therefore, persist regarding how to implement process-tolerant interface chemistries, develop in situ diagnostics capable of correlating interfacial properties with optical performance, and define reproducible surface engineering protocols compatible with pilot-line and foundry-scale manufacturing. Addressing these challenges is essential for transitioning surface-engineered photonic sensors from proof-of-concept demonstrations toward quantitatively reliable and application-ready sensing platforms.

## 8. Outlook and Future Directions

Future advances in integrated photonic sensing are expected to increasingly rely on an interface-first design paradigm in which surface and interface properties are treated as coequal design variables alongside waveguide geometry and material platform [175,176,177]. Rather than optimizing photonic structures independently and subsequently applying functional coatings, emerging approaches emphasize the simultaneous design of optical mode profiles, field localization, and interfacial composition [178]. Such co-design strategies enable deliberate control over the spatial overlap between guided modes and engineered interfaces, allowing sensitivity enhancement while minimizing excess optical loss and preserving spectral coherence [179,180]. This shift is particularly relevant for resonant and interferometric architectures, where interface-induced scattering and absorption directly constrain achievable quality factors and detection limits.

Progress in materials integration is also likely to expand the functional design space available to photonic sensors [181]. Hybrid interfaces incorporating two-dimensional materials, ultrathin oxides, and molecularly engineered dielectric layers offer opportunities to tailor refractive index modulation, surface adsorption kinetics, and charge-transfer interactions at length scales comparable to the evanescent field decay [30,171,182]. However, realizing these benefits in practice will require improved control over interface uniformity, defect density, and environmental stability, as well as a deeper understanding of how electronic and chemical processes at interfaces couple to optical noise and drift. Addressing these challenges will necessitate tighter integration between surface chemistry, materials science, and photonic modeling.

Equally important will be advances in scalable fabrication and metrology that enable reproducible interface engineering at the wafer scale [183,184,185]. In situ and operando characterization techniques capable of correlating interfacial properties with optical performance over time are expected to play a critical role in identifying dominant degradation and variability mechanisms. In parallel, the adoption of standardized benchmarking protocols that incorporate normalized sensitivity metrics, noise characterization, and long-term stability assessments will be essential for comparing interface strategies across platforms [186]. Together, these developments will determine whether surface-engineered photonic sensors can transition from laboratory demonstrations to quantitatively reliable and manufacturable technologies for real-world sensing applications [187].

## 9. Conclusions

Surface and interface engineering has emerged as a central determinant of performance in integrated photonic sensors, redefining how sensitivity, selectivity, stability, and scalability are achieved in practice. As photonic architectures mature and approach the limits set by optical confinement and fabrication tolerances, further advances increasingly depend on controlling the nanoscale region where guided optical modes interact with their environment. This review has shown that the sensing interface is not a passive boundary but an active element that governs light–matter interaction strength, noise behavior, and long-term reliability. Engineering in this region enables access to sensing mechanisms beyond bulk refractive index changes, including molecular recognition, adsorption-driven modulation, and charge-transfer-assisted transduction, while simultaneously introducing new constraints that must be managed through careful co-design.

A key insight emerging from this work is that improvements in surface sensitivity cannot be evaluated independently of optical degradation and interfacial stability. Functional layers, nanomaterials, and hybrid interfaces alter modal confinement, propagation loss, and spectral response in ways that are highly architecture-dependent. As demonstrated across resonant and interferometric platforms, identical surface strategies can yield fundamentally different performance outcomes depending on how optical fields are distributed and interrogated. These technological aspects highlight the need for interface-aware benchmarking frameworks that account for interaction length, optical loss, noise characteristics, and temporal drift, rather than relying solely on nominal sensitivity metrics. Such normalized and noise-aware figures of merit are essential for identifying genuine performance gains attributable to interface engineering rather than changes in device geometry or interrogation conditions.

The review underscores that long-term stability and reproducibility represent critical barriers to the practical deployment of surface-engineered photonic sensors. Chemical evolution of functional interfaces, nanoscale nonuniformity in surface coverage, and variability introduced during fabrication and integration can dominate sensor performance over extended operation times. These effects are amplified in high surface sensitivity and high-quality factor devices, where even sub-nanometer variations in interface properties can induce measurable drift and spectral distortion. Addressing these challenges requires a shift toward process-tolerant interface chemistries, wafer-scale functionalization strategies, and metrology capable of correlating interfacial properties with optical performance in situ.

Looking forward, the continued evolution of integrated photonic sensing platforms will depend on adopting an interface-first design paradigm in which surface engineering is considered an intrinsic component of device architecture rather than a post-fabrication modification. Advances in hybrid material systems, scalable deposition techniques, and standardized performance evaluation will be essential for translating laboratory demonstrations into robust sensing technologies suitable for real-world environments. By integrating surface chemistry, materials science, and photonic engineering within a unified framework, future photonic sensors can achieve not only higher sensitivity but also the stability, reproducibility, and manufacturability required for widespread adoption.

## Figures and Tables

**Table 1:** Impact of surface and interface engineering on integrated photonic sensing.

Interface Strategy	Key Mechanism	Primary Benefit	Main Trade-Off	Most Relevant Architectures
Bare oxide/native surface [74]	Evanescent-field refractive index perturbation	Low optical loss; baseline sensitivity	Poor selectivity; drift	Rib/strip waveguides, interferometers
Self-assembled monolayers (SAMs) [75,76]	Local refractive index changes from molecular binding	High selectivity; minimal thickness	Limited long-term stability	Ring resonators, photonic crystals
Polymer coatings [77,78]	Enhanced adsorption volume	Increased capture efficiency	Absorption; mode delocalization	Strip/slot guides, MZIs
Atomic layer deposition (ALD) oxide overlayers [79]	Interface smoothing and passivation	Reduced scattering loss; improved reproducibility	Sensitivity dilution	High-Q resonators
Two-dimensional materials [80,81]	Charge transfer; absorption modulation	Additional transduction mechanisms	Increased loss; transfer defects	Slot/exposed-core waveguides
Hybrid interfaces [82,83]	Coupled optical–chemical effects	Balanced performance between sensitivity and stability	Fabrication complexity	Advanced resonant sensors
Anti-fouling layers [32,84]	Suppression of nonspecific adsorption	Improved stability and specificity	Reduced sensitivity	Biosensors in complex media

**Table 3:** Comparative benchmarking metrics and trade-offs in surface- and interface-engineered photonic sensors.

Benchmarking Aspect	Reported Metric	Key Limitations	Recommended Metric
**Sensitivity [19,148,149,150]**	Bulk sensitivity (e.g., resonance wavelength shift per refractive-index units)	Depends on device geometry, interaction length, and readout noise	Sensitivity normalized by interaction layer thickness and optical loss/noise contributions
**Limit of detection (LOD) [151,152,153,154]**	Minimum detectable change in refractive index or analyte concentration	Often derived from sensitivity alone, neglecting noise characteristics	Noise-limited LOD defined relative to baseline standard deviation and confidence level
**Optical loss [43,155,156]**	Propagation/insertion loss	Reported independently of sensing performance, obscuring trade-offs	Sensitivity normalized per unit optical loss
**Resonance** **characteristics [101,157,158,159]**	Quality factor or linewidth	High Q does not ensure low LOD; performance is often limited by phase noise and environmental instability	LOD evaluated as a function of linewidth, contrast, and noise
**Signal-to-noise** **ratio (SNR) [160,161]**	Peak shift or intensity change	Does not account for temporal drift or environmental noise	SNR evaluated over relevant averaging times
**Temporal stability [162,163]**	Short-term repeatability	Limited time window; does not capture long-term drift	Allan variance and drift rate analysis
**Repeatability and robustness [164]**	Single-cycle repeatability	Does not account for fouling or surface degradation	Multi-cycle reproducibility under controlled conditions

## Data Availability

No new data were created or analyzed in this study.
